# Identifying the key barriers, facilitators and factors associated with cervical cancer screening attendance in young women: A systematic review

**DOI:** 10.1177/17455057251324309

**Published:** 2025-03-13

**Authors:** Sonia Shpendi, Paul Norman, Jilly Gibson-Miller, Rebecca Webster

**Affiliations:** 1 School of Psychology, University of Sheffield, Sheffield, UK; 2 School of Education, University of Sheffield, Sheffield, UK

**Keywords:** cervical cancer, screening, behaviour, young women, barriers, facilitators, review, pap smear

## Abstract

**Background:**

Cervical cancer (CC) results in around 604,00 new cancer cases yearly and is caused by the human papillomavirus (HPV). Uptake rates for both the HPV vaccination and screening have been decreasing over recent years, particularly in young women, whilst CC remains a concern for both low- and high-income countries.

**Objectives:**

To highlight the key barriers and facilitators of CC screening attendance in young women and to identify the factors associated with their CC screening behaviour, to inform interventions to increase screening rates.

**Design:**

Systematic review.

**Data sources and methods:**

A systematic review was conducted using Scopus, Web of Science, MEDLINE, PsycINFO/PyscARTICLES and CINAHL. The review included primary qualitative, quantitative and mixed-method studies that reported barriers, facilitators and factors associated with current CC screening behaviours in women aged 30 or below. Outcomes were summarised narratively. Risk of bias was conducted for individual studies using the Mixed-Method Appraisal Tool.

**Results:**

Among the 106 studies included in the review, the most frequently reported barriers were financial constraints (*n* = 36), embarrassment (*n* = 35) and low accessibility to obtaining screening (*n* = 34). The most frequently reported facilitators were knowledge of CC (*n* = 12), healthcare provider recommendations (*n* = 11) and communication with friends (*n* = 11). Age (older), marital status (in a relationship) and sexual activity (active) were key factors associated with attendance at screening. Studies also highlighted that those vaccinated were more likely to have screened than those not vaccinated against HPV.

**Conclusion:**

These unique factors represent potential targets for interventions to increase CC screening attendance in young women. Future research could benefit from employing strong theoretical frameworks, such as the COM-B model of behavioural change, to categorise and gain further insight into the contributing factors affecting CC screening attendance.

**Registration:**

PROSPERO CRD42022324948.

## Introduction

There are around 604,000 new cases of cervical cancer (CC) a year globally.^1^ CC has been linked to several risk and lifestyle factors, such as sexual history and smoking,^2^ with persistent human papillomavirus (HPV) infection remaining one of the most common causes of CC.^3^ In 2018, the World Health Organisation called for coordinated global action to eliminate CC, ensuring that all girls are vaccinated against HPV and that at least 70% of women be screened by the age of 35.^4^ The last few decades have seen a decline in mortality rates of CC, with cervical screening programmes and HPV immunisation programmes supporting this.^5,6^ However, uptake rates for both the HPV vaccination and screening have been decreasing over recent years, particularly in young women,^7–9^ whilst CC remains a health concern for both low- and high-income countries.^10–12^

Although cervical screening guidelines vary slightly across different regions, they typically recommend that screening should start between the ages of 20 and 30 years old. However, first-time attendees and young women often face challenges in attending CC screening, such as difficulties making appointments, time constraints and perceived low priority.^13,14^ Previous research has also indicated the positive impact of past behaviour on intention and future health behaviours,^15,16^ underscoring the importance of initial screening attendance and experiences and the effect on subsequent screening attendance. In order to improve screening rates, it is important to identify the key facilitators of, and barriers to, CC screening in young women, who are attending screening for the first time.

A previous systematic review^17^ identified various barriers and facilitators to CC screening in women under 35 years old. Common barriers included: lack of knowledge/awareness, negative perceptions of testing and practical barriers. Common facilitators included increasing knowledge and awareness, trusting relationships with healthcare providers and specific improvements to overcome logistical barriers to screening. However, a gap remains regarding understanding the full range of factors associated with screening attendance in this age group, including socio-demographic or psychological factors, as well as the identified perceived barriers/facilitators themselves. In addition, initial cervical screening is most commonly recommended to 25–29 year olds, across both high- and low-income countries^18^; hence, focusing on those 30 years old and under is likely to better capture initial screening behaviours. The previous review^17^ also only included studies that explicitly mentioned an age cut-off in the title/abstract, which may have resulted in the exclusion of potentially relevant studies. Moreover, as a large portion of women now reaching the screening age are likely to have been vaccinated (or offered the vaccine),^8,19^ it is important to also assess the possible impact of the immunisation programme on first-time screening behaviours.

The current systematic review therefore aims to systematically categorise a wide range of factors, including vaccination status, which may impact screening in young women who are first-time screening participants.

## Methods

The reporting of this review adheres to the standards for the Preferred Reporting Items for Systematic Reviews and Meta-Analyses (PRISMA).^20^ Methods of the analysis and inclusion criteria were specified in a pre-registered protocol (PROSPERO CRD42022324948). PRISMA guidelines were followed when preparing this article.^20^

### Search strategy

SS and RW tested a variety of different search strategies to find balance in the specificity and sensitivity of the terms. These were finalised in discussion with JGM. The final search strategy used terms and associated words for ‘HPV’, ‘CC’ and ‘Screening’ (see Supplemental materials). The search strategy was modified for each specific database due to differences in MeSH terms and Boolean operators.

### Searches

The following electronic databases were searched: Web of Science, MEDLINE, Scopus, PsycINFO and PyscARTICLES) and CINAHL. No grey literature was searched.

### Review process

SS and RW tested the screening process for one database prior to the full database search. SS carried out a full search on 20 December 2021 (updated 1 June 2023). The searches were combined using Mendeley with duplicates identified and deleted. Firstly, titles and abstracts were screened for mentions of barriers, facilitators and/or factors associated with cervical screening in an under 30-year-old majority sample (i.e. >50%). If this was not clear in the abstract, the study was taken to full-text review. Second, all full-text articles were screened in relation to the exclusion/inclusion criteria. Authors were contacted directly in instances where the full-text article was not readily available. If no response was received after two contact attempts, studies were excluded. RW screened 15 of the full-text articles screened by SS to ensure consistency in inclusion. Any disagreements were discussed with JGM. Similarly, if the age of the sample was not clear in the reported study, authors were contacted directly, and studies were included or excluded accordingly. Forward and backward citation searches were also carried on articles that met the inclusion criteria.

### Selection process

Studies were eligible for inclusion if they met the following criteria:

- *Population:* Females aged 30 years old or under.
- *Exposure:* CC screening including invitation and/or attendance behaviour.
- *Outcome:* The study reported data on barriers AND/OR facilitators to CC screening AND/OR factors associated with CC screening.
- *Study design:* Both qualitative and quantitative studies were eligible. Quantitative studies could be of any design. Articles that did not report on original data, for example, reviews or editorials, were excluded.
- *Other limiters:* Published in the English language.

### Data extraction

Data from the final set of studies were extracted by SS and included: author (year of publication), country, design, population description (sample size and sample description), age, type of screening, outcomes, reported facilitators, reported barriers, factors associated with screening behaviour.

### Quality assessment

The quality of included studies was assessed using the Mixed-Method Appraisal Tool (MMAT).^21^ This was used for all study designs included (quantitative, qualitative and mixed methods). The original ‘yes’, ‘unclear’ or ‘no’ answers were used. Eleven (10%) of included studies were quality assessed by a second researcher and scores agreed with SS.

### Data analysis

Heterogeneity in study designs and outcomes was expected; therefore, we did not plan for any meta-analyses and instead used a narrative synthesis. As there is no consensus on the best way to carry out a narrative synthesis for systematic reviews,^22^ we used a weight-of-evidence approach in which the quality of studies was considered when assessing the strength of evidence. The narrative synthesis reports on study characteristics (e.g. author, year, country of origin and setting), study design (e.g. design, outcomes measures used and methodology included), participant characteristics (e.g. age and sample size) and results relevant to the chosen outcomes.

## Results

### Search results

Searches yielded a total of 26,120 articles, of which 12,978 articles were excluded after removal of duplicates and an additional 44 articles were identified through reference list searches and 1929 articles through forward citation searches, resulting in 15,115 articles for title and abstract screening. Following this initial screening process, 692 full-text articles were screened. In total, 106 articles were included in the systematic review.

**Figure 1.**
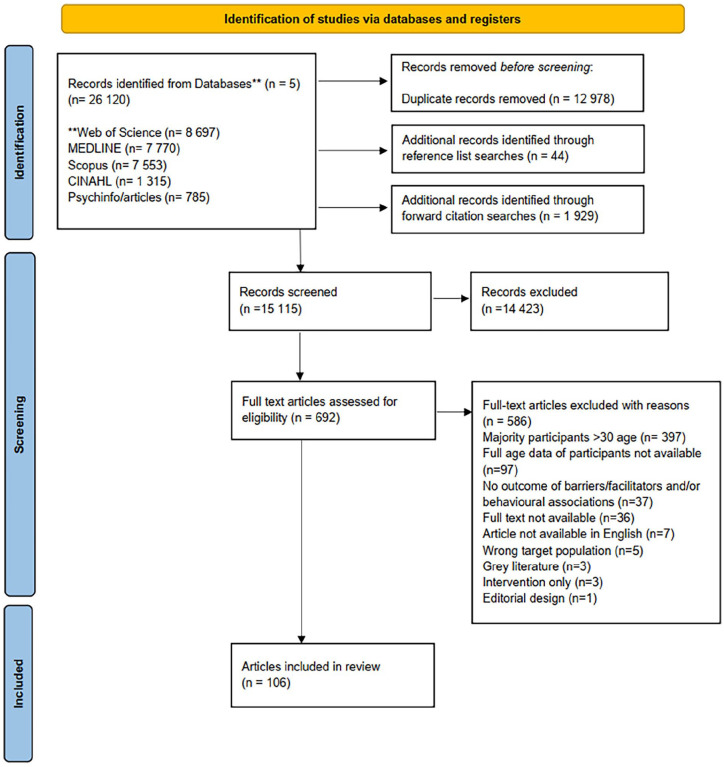
PRISMA flow diagram. **Individual databases used for searches.

Articles were excluded for several reasons (*n* = 586) including, the majority of participants being over 30 years old (*n* = 397), accurate data regarding participants’ age not available (*n* = 97), not reporting barriers, facilitators or factors associated with screening (*n* = 37), full-text articles not being available (*n* = 36), no English version being available (*n* = 7), the inclusion of the wrong target population (e.g. male participants) (*n* = 5), being an intervention based only study (*n* = 3), being grey literature (*n* = 3) and not being an original research article (*n* = 1) (see Figure 1).

### Article characteristics

Studies were published between 1996 and 2024. The majority employed quantitative and observational methods (*n* = 85), including questionnaires and surveys (*n* = 74), cohort studies (*n* = 5), case–control studies (*n* = 1), mixed methods (*n* = 3), quasi-experimental (*n* = 1) and randomised controlled trials (RCTs) (*n* = 1). The mixed-method studies comprised a cross-sectional survey with interviews (*n* = 2) as well as a cross-sectional survey with focus groups (*n* = 1). Other studies used qualitative methods (*n* = 10; interviews *n* = 2, focus groups *n* = 6 and both *n* = 2), utilised secondary data (*n* = 7) or were retrospective (*n* = 4).

The largest portion of the studies were conducted in Africa (*n* = 44; Nigeria, Ethiopia, Malawi, South Africa, Bhutan, Ghana, Kenya, Uganda, Zimbabwe and Lesotho); 30 were conducted in North America (United States, Canada and Dominica); 19 in Asia (India, Singapore, Japan, Malaysia, Nepal, Saudi Arabia, Pakistan, Thailand, Korea and Oman); eight in Europe (United Kingdom, Whales, Sweden and Greece); five in Oceania (Australia) and one in South America (Brazil).

The sample size of included studies ranged from 12 to 699,686. Almost half of included studies targeted student populations (42/106, 39.62%). Most studies discussed Pap smears specifically (75/106, 70.75%), whereas 11 articles also discussed Visual Inspection with Acetic Acid (VIA), four articles HPV testing, two Visual Inspection with Lugol’s Iodine (VILI) and one article Liquid-Based Cytology, High Vaginal Swab and Endocervical Swab, as well as Pap smears. Six studies focused solely on VIA methods of screening. Twenty-three studies did not specify a particular type of screening. Sixty-six studies included details on barriers to screening, 18 on facilitators of screening and 74 on factors associated with screening. See Table 1 for full study characteristics and Table 2 for summary of reported themes.

**Table 1.** Study characteristics.

Reference (year published)	Country	Study design	Sample size; sample description	Average age (SD)	Type of screening	Outcome(s)
Abiodun et al. (2014)^23^	Nigeria	Quasi-experimental	614 Women	NR (majority 25–34, EXP 72.3% and CON 70.3%)	VIA	Barriers
Abotchie et al. (2009)^24^	Ghana	Cross-sectional survey	140 University students	NR (age range 18–35, majority 21–25, 66.2%)	Pap smear	Barriers
Ackerson et al. (2008)^25^	USA	Qualitative interviews	7 African American women	28 (SD NR)	Pap smear	Barriers, facilitators
Ackerson et al. (2014)^26^	USA	Cross-sectional survey	67 Undergraduate female nursing students	23 (4.78)	Pap smear	Barriers, factors associated
Akpo et al. (2016)^27^	Dominica	Cross-sectional survey	100 Female medical students	NR (age range 15–29)	Pap smear	Barriers
Akujobi et al. (2008)^28^	Nigeria	Cross-sectional survey	220 University students	23.8 (SD NR, age range 17–39)	Pap smear	Barriers
Al-Naggar et al. (2010)^29^	Malaysia	Cross-sectional survey	285 University students	20.9 (1.89)	Pap smear	Barriers
Alwahaibi et al. (2017)^30^	Oman	Cross-sectional survey	494 Outpatients, hospital staff and students	NR (age range NR, majority 20–30, 68.6%)	Pap smear	Barriers, facilitators, factors associated
Alwahaibi et al. (2018)^31^	Oman	Cross-sectional survey	494 Outpatients, hospital staff and students	NR (age range </20 majority 20–29, 68.2%)	Pap smear	Factors associated
Anaman et al. (2017)^32^	Australia	Cross-sectional survey	254 African-born women	NR (majority 21–29, 52%)	Pap smear	Facilitators, factors associated
Aniebue et al. (2010)^33^	Nigeria	Cross-sectional survey	394 Hostel residents	23.8 (3.8)	Pap smear	Barriers, facilitators, factors associated
Anikwe et al. (2021)^34^	Nigeria	Cross-sectional survey	325 University students	NR (age range majority 21–25, 47.45%)	Unspecified	Barriers, factors associated
Annan et al. (2019)^35^	Ghana	Cross-sectional survey	200 Undergraduate students	20.4 (1.96)	Unspecified	Factors associated
Argaw et al. (2022)^36^	Ethiopia	Cross-sectional survey	385 Sex workers	29.3 (5.5)	Pap smear, VIA	Barriers, factors associated
Aweke et al. (2017)^37^	Ethiopia	Cross-sectional survey	583 Childbearing women	NR (median 28)	VIA	Barriers, factors associated
Ayeni et al. (2023)^38^	Nigeria	Cross-sectional survey	362 Women of reproductive age	25.19 (7.18)	Unspecified	Barriers
Ayinde et al. (2004)^39^	Nigeria	Cross-sectional survey	421 Undergraduate students	23.6 (3.6)	Pap smear	Barriers, factors associated
Bakogianni et al. (2012)^40^	Greece	Cross-sectional survey	472 Students	21.3 (5.18)	Pap smear	Barriers
Bayu et al. (2016)^41^	Ethiopia	Cross-sectional survey	1186 Women living in Mekelle zone	31.3 (9.3)	VIA	Barriers, factors associated
Bammeke et al. (2014)^42^	Nigeria	Cross-sectional survey	100 Women of reproductive age	NR (majority 26–30, 45%)	Unspecified	Barriers
Beer et al. (2014)^43^	UK	Secondary data analysis	30,882 Residents in Wales	NR (age range 22–24)	Unspecified	Factors associated
Bekele et al. (2022)^44^	Ethiopia	Cross-sectional survey	687 Female students	20.5 (3)	VIA	Facilitators, factors associated
Binka et al. (2016)^45^	Ghana	Cross-sectional survey	410 Students	NR (age range majority 20–29, 61%)	Unspecified	Factors associated
Black et al. (2011)^46^	Canada	Qualitative focus groups	80 Attendants of university health clinics, shopping centres and community centres serving young women	NR (age range 20–29)	Pap smear	Barriers, facilitators
Boone et al. (2016)^47^	USA	Retrospective matched-pair cohort study	2246 HPV vaccinated and unvaccinated women	NR (age range 14–26)	Unspecified	Factors associated
Budd et al. (2014)^48^	Australia	Cross-sectional records review	NR: Young women	NR (age range 20–34)	Pap smear	Factors associated
Burak and Meyer (1998)^49^	USA	Cross-sectional survey	400 Undergraduate students	19.1 (SD NR)	Pap smear	Barriers, factors associated
Byrd et al. (2004)^50^	USA	Cross-sectional survey	189 Hispanic women	21 (SD NR; age range 18–25)	Pap smear	Factors associated
Changkun et al. (2022)^51^	India	Secondary data analysis	699,686 Women from the NFHS	NR (majority 15–29, 51.9%)	Unspecified	Factors associated
Chao et al. (2017)^52^	USA	Retrospective cohort study	27,352 KPSC members	NR (age range 25–30)	Pap smear	Factors associated
Cooper et al. (2018)^53^	Australia	Qualitative interviews	12 University students	21 (SD NR, age range 18–25)	Pap smear	Barriers, facilitators
Deresse and Aebra (2018)^54^	Ethiopia	Cross-sectional survey	821 Women	26.07 (5.57)	Pap smear, VIA	Barriers
Dhendup and Tshering (2014)^55^	Bhutan	Cross-sectional survey	559 Graduate students	23.43 (2.73)	Pap smear	Barriers, factors associated
Dozie et al. (2021)^56^	Nigeria	Cross-sectional survey	375 Female undergraduates	NR (age range 16–29)	Pap smear, VILI, VIA	Barriers, factors associated
Easwaran et al. (2023)^57^	Saudi Arabia	Cross-sectional survey	185 Pharmacy students	19.77 (6.71)	Unspecified	Barriers, facilitators, factors associated
Eiser and Cole (2002)^58^	UK	Cross-sectional survey	70 Students	21.6 (1.14)	Pap smear	Barriers
Enyan et al. (2022)^59^	Ghana	Cross-sectional survey	431 Muslim women	30.9 (10.4)	Unspecified	Facilitators, factors associated
Gebisa et al. (2022)^60^	Ethiopia	Cross-sectional survey	414 Women attending health facilities	NR (age range 18–49)	Unspecified	Barriers, factors associated
Gebreegziabher et al. (2016)^61^	Ethiopia	Cross-sectional survey	225 Female nurses	NR (median 28)	Pap smear	Barriers, factors associated
Gebregziabher et al. (2019)^62^	Ethiopia	Cross-sectional survey	344 Undergraduate students	23.67 (2.83)	Pap smear	Barriers, factors associated
Gebru et al. (2016)^63^	Ethiopia	Cross-sectional survey	643 Married women	NR (majority 20–24, 27.1%)	Pap smear	Factors associated
Gelassa et al. (2023)^64^	Ethiopia	Cross-sectional survey	213 Women attending health faculties	32.2 (13.8)	Pap smear, VIA	Barriers, factors associated
Getaneh et al. (2021)^65^	Ethiopia	Cross-sectional survey	403 Undergraduate students	21 (1.5)	Pap smear	Barriers
Guo et al. (2017)^66^	USA	Secondary data analysis	5416 Respondents of NIHS survey	NR (age range 21–30)	Pap smear	Factors associated
Hauwa et al. (2021)^67^	Nigeria	Cross-sectional survey	230 Women	NR (majority 25–29, 30%)	Pap smear, VIA	Factors associated
Head and Cohen (2012)^68^	USA	Qualitative focus groups and interviews	19 Women	NR (age range 20–26)	Pap smear	Barriers, facilitators
Hirth et al. (2016)^69^	USA	Retrospective cohort study	24,964 Female health records	NR (age range 19–21)	Pap smear	Factors associated
Hoque et al. (2014)^70^	South Africa	Cross-sectional survey	440 University students	20.39 (1.71)	Pap smear	Factors associated
Ibekwe (2015)^71^	Ethiopia	Comparative cross-sectional survey	200 Clinical nursing students	DELSUTH 24.2 (2.6) and UBTH 23.2 (2.9)	Unspecified	Barriers
Ilika et al. (2016)^72^	Nigeria	Descriptive cross-sectional survey	342 Undergraduate students	NR (majority 20–29, 97.7%)	Pap smear, HVS, ES, VIA	Barriers, factors associated
Isabirye et al. (2022)^73^	Zimbabwe	Secondary data analysis	9955 Women from the Zimbabwe demographic 7 health survey	NR (age range 15–49)	VIA	Factors associated
Isara et al. (2013)^74^	Nigeria	Descriptive cross-sectional survey	230 Medical students	20 (1.4)	Pap smear	Barriers, factors associated
Jemal et al. (2023)^75^	Ethiopia	Mixed methods	241 Female health workers	Nr (age range majority </30, 72%)	Unspecified	Barriers, factors associated
Jubelirer et al. (1996)^76^	USA	Cross-sectional survey	279 10th-grade students	NR (age range 14–18)	Pap smear	Barriers
Kabirir and Komuhangi (2021)^77^	Uganda	Cross-sectional survey	355 Female undergraduate students	NR (majority 21–25, 60.6%)	Pap smear, VIA	Barriers, facilitators, factors associated
Kahn et al. (1999)^78^	USA	Qualitative focus groups and interviews	27 Adolescents receiving care from a children’s hospital	Focus group 17.6 (2.3); interviews 18.7 (1.9)	Pap smear	Barriers
Kakubari et al. (2020)^79^	Japan	Cross-sectional survey	618 Residents of Japan	NR (age range 20–21)	Unspecified	Barriers, factors associated
Kaneko (2018)^80^	Japan	Cross-sectional survey	700 Unmarried Japanese females	26 (SD NR)	Pap smear	Factors associated
Karena et al. (2024)^81^	India	Cross-sectional survey	97 Female nursing staff	NR (age range 20–29)	Pap smear, VIA	Barriers, facilitators
Kim et al. (2016)^82^	USA	Nested case-control study	10,204 Screened residences of Alberta	NR (age range 18–33)	Pap smear	Factors associated
Kitchener et al. (2018)^83^	UK	Cluster RCT	10,126 First screening invitation recipients	NR (age range 20–24.5)	Unspecified	Factors associated
Kreusch et al. (2018)^84^	Sweden	Cohort study	261,434 Residents of Sweden	NR (age range 24–27)	Unspecified	Factors associated
Langille and Rigby (2006)^85^	Canada	Cross-sectional survey	1090 Female students	16.6 (0.1)	Unspecified	Factors associated
Lee and Lee (2017)^86^	USA	Qualitative focus groups	16 Korean immigrant women	26 (SD NR, age range 21–29)	Pap smear	Barriers
Lee et al. (2015)^87^	USA	Cross-sectional survey	164 Hmong American immigrant women	30 (SD NR, age range majority 21–29, 59.8%)	Pap smear	Factors associated
Letuka and De Wet (2018)^88^	Lesotho	Cross-sectional records review	1542 Residents of Lesotho	NR (age range 15–19)	Pap smear	Factors associated
Mather et al. (2012)^89^	Australia	Cross-sectional survey	193 Psychology university students	Vacc 19.2 (2.05); Unvac 19.5 (2.10)	Pap smear	Factors associated
Miyoshi et al. (2021)^90^	Japan	Cross-sectional survey	435 Japanese members of an internet survey panel	NR (age range 18–19)	Unspecified	Factors associated
Moreira et al. (2006)^91^	Brazil	Cross-sectional survey	204 Women in waiting room of a gynaecological clinic	20 (2)	Pap smear	Barriers
Moudatsou et al. (2022)^92^	Greece	Cross-sectional survey	100 Female students	22.2 (2)	Pap smear	Barriers, factors associated
Mpachika-Mfipa et al. (2023)^93^	Malawi	Cross-sectional survey	482 Women	NR (age range majority 18–24, 42.5%	VIA	Factors associated
Mpachika-Mfipa et al. (2022)^94^	Malawi	Cross-sectional survey	482 Women	NR (confirmed via author)	Unspecified	Factors associated
Najem et al. (1996)^95^	USA	Cross-sectional survey	3343 Senior high school students	NR (age range 13–20)	Pap smear	Barriers, factors associated
Natae et al. (2021)^96^	Ethiopia	Cross-sectional survey	392 Women	NR (majority 20–29, 58.2%)	VIA	Barriers, factors associated
Ndikom and Ofi (2012)^97^	Nigeria	Qualitative focus groups	82 Attenders of various health facilities	27.6 (4.5)	Pap smear	Barriers
Ngari et al. (2021)^98^	Kenya	Cross-sectional survey	80 Women	NR (age range 15–25)	Pap smear, VILI, VIA	Barriers
Ogbechie et al. (2012)^99^	USA	Cross-sectional survey	66 Visitors of obstetrics and gynaecology clinic	22.2 (1.9)	Pap smear	Factors associated
Ogbonna (2017)^100^	UK	Cross-sectional survey	186 Sub-Saharan African students	NR (age range </18, majority 18–24, 56.5%)	Pap smear	Barriers
Osei et al. (2021)^101^	Ghana	Qualitative focus groups	35 Community women	NR (age range 19–60, majority 19–29, 71.4%)	Pap smear	Barriers, facilitators
Oshima and Maezawa (2013)^102^	Japan	Qualitative focus groups	15 Japanese university students	NR (age range 20–22)	Pap smear	Barriers
Owoeye and Ibrahim (2013)^103^	Nigeria	Descriptive cross-sectional survey	360 University staff and students	23.65 (5)	Pap smear, liquid-based cytology and HPV DNA	Barriers, facilitators, factors associated
Park et al. (2023)^104^	South Korea	Secondary data analysis	17,730 Married immigrant women	NR (age range 20–29)	Pap smear	Factors associated
Paynter et al. (2015)^105^	USA	Retrospective cohort study	2308 Attenders of a medical centre	20.6 (0.09)	Unspecified	Factors associated
Pegu et al. (2017)^106^	India	Descriptive cross-sectional survey	34 Nursing staff	25 (SD NR)	Pap smear	Barriers
Pengpid and Peltzer (2014)^107^	Multicounty	Cross-sectional survey	9194 Undergraduate students	20.9 (2)	Pap smear	Factors associated
Reiter and McRee (2014)^108^	USA	Cross-sectional survey	418 Members of the LGBTQ community	23.8 (1.7)	Pap smear, HPV self-testing	Barriers, factors associated
Rosita et al. (2023)^109^	India	Cross-sectional mixed methods	125 Female nurses	NR (majority 20–24, 76.8%)	Pap smear	Barriers, facilitators
Sadler et al. (2013)^110^	UK	Qualitative focus groups	31 Women registered at a general practice	NR (age range 17–25)	Unspecified	Barriers, facilitators
Sauer et al. (2015)^111^	USA	Cross-sectional records review	7341 Young women	NR (age range 21–30)	Pap smear	Factors associated
Sauvageau et al. (2021)^112^	Canada	Cross-sectional survey	1475 Young adults	NR (age range 17–29)	Pap smear	Factors associated
Seay et al. (2022)^113^	USA	Secondary data analysis	34,141 Active female US military service members	NR (majority 20–29, 79.4%)	Pap smear, HPV testing	Factors associated
Shand et al. (2010)^114^	Australia	Cross-sectional survey	274 Residents of Australia	21.75 (2.14)	Pap smear	Factors associated
Shin et al. (2022)^115^	Korea	Secondary data analysis	3925 Korean women	NR (majority 20–29, 51%)	Pap smear	Factors associated
Shin et al. (2021)^116^	Korea	Mixed methods	26 Female university students	21.92 (1.26)	Pap smear	Barriers
Singh et al. (2022)^117^	India	Cross-sectional survey	100 Nursing staff	NR (majority 26–30, 48%)	Pap smear	Barriers
Singh et al. (2012)^118^	India	Descriptive cross-sectional survey	133 Nursing staff	27.82 (3.85)	Pap smear	Barriers, factors associated
Tadesse et al. (2022)^119^	Ethiopia	Cross-sectional survey	667 Female students	NR (majority 15–20, 85.2%)	Pap smear, VIA	Barriers, facilitators
Tang et al. (1999)^120^	USA	Cross-sectional survey	206 Undergraduate and graduate students	19 (20)	Pap smear	Barriers, factors associated
Tay et al. (2015)^121^	Singapore	Cross-sectional survey	1622 Staff nurses	NR (age range >/25, majority <30, 56.9%)	Unspecified	Barriers, facilitators, factors associated
Tesfaye et al. (2022)^122^	Ethiopia	Cross-sectional survey	393 Women hospital employees	NR (majority 25–29, 51.6%)	Unspecified	Barriers, factors associated
Thapa et al. (2018)^123^	Nepal	Cross-sectional survey	360 Women	30.13 (10.4)	Pap smear, HPV test, VIA	Barriers, factors associated
Ugonwanyi et al. (2014)^124^	Thailand	Cross-sectional survey	172 Female international students	24.4 (5.5)	VIA	Barriers, Factors associated
Wellensiek and Yamarat (2002)^125^	South Africa	Cross-sectional survey	750 Attenders of a hospital, medical students and student nurses	NR (majority </40, 60.6%, </30 46.41%)	Pap smear	Barriers, factors associated
Yi (1998)^126^	USA	Cross-sectional survey	207 Vietnamese university students	22.7 (3.4)	Pap smear	Factors associated
Yoo et al. (2011)^127^	USA	Cross-sectional survey	304 Korean American, Vietnamese American and Filipino American women	20.82 (SD NR)	Pap smear	Factors associated
Zaidi et al. (2021)^128^	Pakistan	Cross-sectional survey	147 Undergraduate students	25 (0.62)	Pap smear	Barriers, factors associated

NR: not reported; EXP: experimental group; CON: Control group; VILI: visual inspection with Lugol’s Iodine; VIA; visual inspection method with acetic acid; HVS: high vaginal swab; ES: endocervical swab; Vacc: HPV vaccinated; Unvac; not HPV vaccinated; DELSUTH: delta state university teaching hospital; UBTH: University Benin Teaching Hospital; KPSC: Kaiser Permanente Southern California; NFHS: National family health survey.

**Table 2.** Summary of themes and sub-themes reported in the results.

Theme	Sub-theme
Reported barriers to screening	Practical barriers
	Negative perceptions and feelings towards CC screening
	Knowledge and misinformation
	Cultural perceptions/biases
Reported facilitators of screening	
Factors associated with screening	Socio-demographic factors
	Vaccination status
	Psychological factors
	Knowledge
	Previous experience

### Quality assessment

The overall quality of the 106 studies were rated as medium, based on the MMAT quality score.^14^ Most studies reported clear aims and objectives and collected data that addressed the research aims. However, lower quality scores were observed for response rates, representativeness and data collection in quantitative studies and for coherence, findings and data collection in qualitative studies. Lack of clarity around qualitative methods of analysis used was the biggest issue among the qualitative studies and mixed-method studies (*n* = 3). Data analysis in quantitative studies that reported factors associated with screening predominately utilised bivariate analysis (e.g. chi-square and binary logistic regression) and multivariate analysis (e.g. multiple logistic regression). However, factors adjusted for in multivariate analysis were not consistently reported in the included studies. See Supplemental materials for further details.

### Reported barriers to screening

About 66 of the 106 studies reported barriers to CC screening. Barriers were grouped into four overarching themes: practical barriers, negative perceptions and feelings towards CC screening, knowledge and misinformation and cultural perceptions/biases (see Tables 3 and 4 in Supplemental materials).

#### Practical barriers

Several practical barriers were reported that directly impacted young women attending CC screening. Most notably, financial constraints were reported in 36 studies, including concerns over the cost of screening and it being ‘too expensive’ (*n* = 35)^23,24,27,29,30,34,38–42,49,53,56,60,61,64,68,71,72,74,76–78,95,97,98,103,106,108,109,119,123,124,128^ and lack of insurance cover,^25^ reported in Malaysia, Oman, Nigeria, Uganda, Kenya, Thailand, Pakistan, Greece, United States, Australia, Ghana, Nigeria, Ethiopia, Dominica, Nepal and India. Low accessibility to obtaining screening was also commonly reported (*n* = 34),^23,24,26,28–30,33,37,39,42,46,53,54,56,58,61,65,71,72,74–79,86,91,95,97,98,100,109,110,123^ including participants not knowing where to get screening (*n* = 19),^24,28,29,33,39,42,46,53,56,61,65,71,72,74,75,77,79,95,109^ inconvenient locations (e.g. too far; *n* = 14)^23,30,37,42,46,54,58,61,77,78,86,97,98,123^ and difficulty getting an appointment (*n* = 12).^42,61,71,72,74,76–78,91,100,109,110^ Other reported barriers included not knowing how to make the appointment (*n* = 5),^26,54,61,77,95^ childcare constraints (*n* = 2)^46,78^ and moving home and not establishing a relationship with local care providers (*n* = 1).^46^ Time constraints were cited in 24 studies, including being ‘too busy’ and having ‘no time’.^28,30,34,36,46,53,61,65,75,77,78,95,100–102,110,116,121^ Some studies also reported that participants noted no desire to dedicate time^37,63,80,92^ and that screening was time-consuming.^26,41,109^

#### Negative perceptions and feelings towards CC screening

Anxieties, fears and embarrassment of the procedure were prominent among young women. In 35 studies, participants cited embarrassment of the procedure as a barrier to attending CC screening.^24,27,29,34,38,39,41,46,49,57,60–62,64,65,71,72,75,76,78,79,91,95,102,103,106,108,110,117–119,121,123,125,129^ This was followed by fear of pain/discomfort (*n* = 28),^24,28–30,41,42,46,49,53,54,57,58,60–62,64,65,74–78,91,103,106,110,121,124^ feelings of vulnerability (*n* = 8)^30,53,54,68,78,81,109,124^ and fear of the procedure (*n* = 4).^64,117,123,129^

Negative feelings regarding the result after screening were also cited in 24 studies. Young people’s embarrassment and/or fear of a positive result,^28,30,34,36,38,46,54–56,60,61,71,72,75–79,81,97,109,118,122,129^ hesitancy to visit a gynaecologist or other healthcare services (*n* = 4),^40,72,79,102^ and being generally worried about screening (*n* = 2)^24,29^ were notable barriers.

Nine studies noted a lack of trust in screening as a barrier,^24,31,39,55,68,72,77,78,110^ stating the test is not useful (*n* = 3)^31,39,72^ and a lack of belief that the purpose is to diagnose cancer.^24^ Previous negative experiences also were cited in reducing young people’s trust in healthcare recommendations to attend screening (*n* = 4).^31,55,77,110^ Ten studies cited young people felt a lack of encouragement by healthcare workers, or in general, to attend screening.^29,42,123,54,77,95,97,102,108,109,117^

#### Knowledge and misinformation

A lack of awareness and knowledge surrounding all aspects of CC screening was cited in 35 studies across Africa, Europe, North America, Oceania and Asia.^23,26,28,30,34,36–39,42,46,54–57,60,64,65,72,78,79,86,95,97,98,101,109,110,116,119,123–125,128,129^ Nine of these studies based in African countries and Oman reported that young people had never heard of Pap smear/screening^28,31,34,36,37,54,55,65,125^ and another two studies from Japan and Ethiopia reported individuals never having heard of CC.^37,79^ Misinformation regarding being HPV vaccinated and no longer needing screening was cited once^40^ and four articles from Greece, United Kingdom, Uganda and Japan cited young age as an inhibiting factor, with views that screening should be done at a later age.^30,77,79,110^

Five studies reported that not being sexually active was a reason for not being screening,^34,79,92,101,121^ as well as concerns of ‘loss of virginity’ due to the nature of screening, reported in three studies from Malaysia, Ghana, Pakistan and India.^24,29,65,128^

Notably, attitudes of fatalism regarding young people’s overall health were reported in 36 studies. A lack of symptoms was cited 24 times,^25,27,30,33,36–38,41,56,57,60,64,65,75,79,81,103,108,117–119,123,124,129^ whereas 11 studies also reported that participants did not believe that cancer affected them.^39,42,61,77–79,81,95,110,117,118^ Five studies reported an overall lack of interest in screening.^23,26,34,65,98,109^ Four studies reported screening as simply not necessary with no further explanation.^28,74,92,121^

#### Cultural perceptions/biases

Cultural biases and/or prejudices against screening were reported in four studies.^54,86,98,120^ However, multiple studies reported specific cultural reasons for not attending screening. Fear of being seen or spoken about was reported in six studies from Nigeria, Uganda, Japan and United States. This included reports of worrying what others might say,^24,102,116^ being afraid of being seen visiting the gynaecologist,^102^ and fear of parents finding out about sexual behaviour.^68,76,78,116^ Spousal and familial roles as barriers to screening were also reported. In eight studies from Malaysia,^29^ Ethiopia,^54,60,61,64,65^ India^109^ and Ghana^24^ young women reported a spouse not allowing attendance to screening as a barrier.

### Reported facilitators of screening

Eighteen studies included reports of facilitators of CC screening (see Tables 5 and 6 in Supplemental materials). Increased knowledge of and belief in CC screening were the most commonly reported facilitators in 12 studies.^25,30,44,53,59,77,81,101,103,109,119,121^ Specific points included the belief that screening reduces risk,^25,77,121^ general awareness,^77,81,101^ understanding the importance of screening^30,77^ and the long-term benefit of screening.^53,77^

Healthcare provider recommendations and reminders were also commonly reported facilitators.^30,33,44,46,59,103,109,110,119,121,130^ Five studies based in Ghana,^101^ Oman,^30^ Nigeria,^103^ Uganda^77^ and India^109^ cited the financial facilitator of CC screening being cost-free, whether in general, at work or during a CC screening awareness month incentive.^101^ One study also cited being able to afford screening as a facilitator of attendance.^77^

Mention of opportunistic reasoning for attending was reported in eight studies,^30,46,57,59,77,81,103,109^ including, during pregnancy,^46^ renewal of oral contraceptives,^46^ when combined with other tests,^81,103^ having enough time during that period^30^ and having a convenient location.^59^

Communication with friends and family was reported in 11studies. Specifically, friends’ encouragement and open conversation around the procedure and topic were recognised as a facilitator in seven studies.^25,30,44,53,77,119,121^ Similarly, maternal involvement in promoting CC screening and as a source of information was noted in three studies.^25,53,68,130^

### Factors associated with screening

Seventy-four studies analysed factors associated with CC screening. These were grouped into four overarching themes: Socio-demographic factors, vaccination status, psychological factors, knowledge and previous experiences (see Tables 7 and 8 in Supplemental materials).

#### Socio-demographic factors

##### Age

Thirty-one studies examined the relationship between age and screening. The majority of the 20 significant relationships indicated an increase in screening attendance with older age,^31,41,85,94,95,108,127,129,51,55,56,62,63,69,73,80^ although some studies reported declining attendance with older age.^36,113,121,122^ Although findings were mostly consistent, it is important to consider the varying quality of studies, as only two studies scored high in quality.^36,96^

##### Marital status

Of the 28 studies that examined marital status, all significant findings reported increased odds of screening in those married or in a relationship when compared to those who are single.^26,30–34,39,55,56,62,77,87,88,107,108,114,118,126^ The quality of these studies was broadly consistent as medium quality, although three studies scored lower quality in sampling,^114^ representation^62^ and response rate.^26^ Only one study scored high in quality.^118^

##### Employment status

Fourteen studies examined employment status and eight reported significant results. Five reported that those working were more likely to attend screening than those unemployed or of housewife status^32,51,73,80,115^ and were 5.9 times more likely to attend screening when working compared with being at school.^45^ Healthcare professionals had higher odds of screening when compared to those in the Air Force^113^ and cleaners,^122^ as well as those working in outpatient wards compared to other wards in a hospital.^61^ Overall quality of these studies was consistent but moderate.

##### Education

Sixteen studies of variable quality examined the effect of level of education and found consistent results. The majority reported increased odds in screening with increased education or years in college,^30,44,56,59,62,64,67,73,75,77,120,125^ with only a few studies reporting equivocal^39,92,123^ or negative results.^88^

##### Ethnicity

Ten studies of variable quality examined ethnicity with six reporting mixed significant results.^94,95,105,108,120,123,127^

##### Residence

Six of nine studies that reported place of residence found this to be a significant factor. Those living in urban areas^51,88,94,104^ or major towns^85^ were more likely to attend screening when compared to those living in rural areas. One study explored differences amongst specific regions of Zimbabwe.^73^ Quality scores were moderate across these studies and findings consistent.

##### Sexual activity

Thirteen studies examined sexual activity. Six studies measured this using age at first sexual activity^41,73,77,88,108,125^ and seven reported on whether the women were sexually active or not.^33,39,62,77,109,114,126^ Almost all significant associations, excluding one,^33^ reported a positive association with being sexually active and attending screening.^39,62,77,107,114,126^ One study also reported that the use of a hormonal contraceptive increased odds of screening compared to those using condoms only or an ineffective method.^85^

Seven of 10 studies reported significant results regarding lifetime sexual partners, indicating pap testing is more common amongst those with more sexual partners compared to those with none.^41,75,77,80,107,108,122^ Findings were mostly consistent across studies with moderate scores in quality.

One study reported on sexual orientation, indicating an increased prevalence of screening amongst those who identify as bisexual compared to those who identify as lesbian.^108^

### Vaccination status

Eleven of 13 studies examining HPV vaccination status reported a significant positive association with being vaccinated.^43,47,52,66,79,80,83,84,90,108,111^ When adjusting for age differences in participants, six out of seven studies also found a similar association.^47,48,66,69,82,111^ When also adjusting for race, three studies reported that vaccination still increased the odds of being screened.^52,66,111^ Evidence and quality of studies reporting vaccination were consistent and moderate, although one study scored low in sampling.^80^

#### Psychological factors

Five out of six studies reported greater perceived benefits and/or prevention orientation was significantly positively associated with screening uptake.^26,49,70,107,120^

Five of six studies examining perceived logistical barriers to screening reported that those who had received screening perceived fewer logistical barriers than those who had not.^26,41,50,70,80^ Likewise, one study also reported that students who had been screened scored higher in self-efficacy than those who had not been screened.^70^ Evidence and quality of studies on perceived benefits and logistical barriers were moderate and consistent.

Eleven studies examined the perceived susceptibility of CC and screening.^26,32,35,41,49,63,70,80,108,121,124^ Only five studies found that greater perceived susceptibility was significantly associated with uptake of screening,^32,41,80,124^ even when comparing LGBTQ+ individuals with heterosexual individuals.^108^

Five studies reported on the perceived severity of CC^32,35,41,49,50^ and two studies reported that increased perceived seriousness of CC alone^35^ or more than other cancers^50^ was significantly associated with increased screening attendance. However, given the small number of significant findings in relation to perceived severity and susceptibility, it is difficult to draw a strong conclusion.

#### Knowledge

Twenty-four studies examined knowledge of CC and pap smear and screening. Sixteen studies reported significant positive associations between knowledge and attending screening.^31,32,35,36,37,41,44,55,59,64,75,93,95,122,128^ One study also reported that increased knowledge of HPV was associated with screening uptake.^108^ The quality and findings of these studies were mostly consistent, including five high-scoring quality articles.^36,59,64,96,122^

#### Previous experiences

Five studies reported significant positive associations between screening and having had a routine check-up and/or visited a gynaecologist.^32,96,108,120,121^

Two studies reported that a previous invitation to screening was positively associated with attending screening compared to those who had not received an invitation.^55,95^ A family member’s previous screening was also positively associated with screening.^95^

Three studies examined having a usual source of care,^32,120,126^ although only one found that having a regular place for care was positively associated with screening.^126^ Although findings were consistent for these factors, it is difficult to draw a strong conclusion based on the small number of findings.

## Discussion

### Main findings

Reported barriers to screening were grouped into four main sub-themes: practical barriers, negative perceptions and feelings towards CC screening, knowledge/misinformation and cultural perceptions/biases. Reported facilitators included healthcare provider recommendations, communication with friends and family and knowledge of CC screening. Factors associated with screening fell into four main areas: socio-demographic factors, vaccination status, psychological factors and previous experiences.

Some themes were prevalent across different countries and areas of the world. For example, accessibility and time-constraints appeared throughout, along with more specific concerns over the location^23,30,37,46,54,58,61,78,86,97,123^ and difficulty getting an appointment.^61,71,72,74,76,78,91,100,109,110^ Financial constraints were the most prominent barrier in countries or regions where free screening programmes were not available. However, a study based in Greece, where a free screening programme is available, also cited cost as a barrier, indicating that there could be other financial cost constraints aside from paying for screening (e.g. transport).^40^ Such barriers may be particularly important for younger women who must juggle work and childcare and may not be as financially stable as their older counterparts. Interventions could therefore target improving accessibility by creating opportunities for screening in convenient locations and times, such as drop-in clinics.^14^ Contrary to previous reviews^17,129^ that suggested an impact of socio-economic status on screening, studies included in the current review did not frequently test for the association between socio-economic status and screening, nor report there being a strong association.

Cultural barriers and concerns surrounding loss of virginity and sex-negative beliefs were not prominent, but fears of being seen or spoken about remained a concern across different countries.^24,76,102^ Additionally, lack of encouragement or communication about CC screening from social circles and health professionals which were often reported by participants likely further enhances these negative perceptions. Furthermore, a previous review found moderate strength of evidence that telephone support increased screening uptake in ethnic minorities.^130^ It is perhaps unsurprising that one of the main reported facilitators among young people was the importance of open communication about screening with friends and family and recommendations from healthcare providers.

Psychological barriers were far more prevalent in the current review in comparison to a previous review.^17^ Feelings of fear and embarrassment surrounding multiple aspects of the screening procedure and fear of the results were the most often reported barriers for young women. The prevalence of fear as a barrier to screening was also highlighted in a previous review of studies based in sub-Saharan Africa.^131^ However, only 14 studies statistically tested the relationship between at least one psychological factor and screening uptake.^24–26,49,50,59,68,70,78,87,97^ Moreover, it is interesting to note that only 11 articles utilised a theoretical framework, with the Health Belief Model (HBM) being the most popular.^24,49,50,59,70,78,97^ The HBM is a health behaviour change model developed to explain and predict health-related behaviours, with a focus on uptake in health services. HBM constructs focus on an individual’s perceptions of the health threat (i.e. perceived susceptibility, perceived severity) and the health actions can prevent it (i.e. perceived benefits, perceived barriers).^132^ Perceived susceptibility, benefits and logistical barriers were most frequently analysed and consistently associated with screening uptake.

A lack of awareness and knowledge surrounding CC and screening was consistently reported as a barrier for young women across countries. This was supported by studies highlighting the positive impact of increased knowledge on screening attendance, and the fact that it was a common self-reported facilitator for those who had attended. Given that CC screening is likely the first invitation or experience of a pelvic exam, it is vital that young people are equipped with a basic knowledge and understanding of the purpose of CC screening.

Reported demographic factors associated with screening highlighted that being in a relationship or married, being older, being sexually active or being vaccinated were significantly associated with screening attendance. Multiple reasons could explain why those vaccinated are attending screening more than those unvaccinated. Despite screening rates declining over the past decade,^133^ the evidence does not suggest that this is likely due to the introduction and success of the HPV vaccination programme. The suggestion that vaccination could result in a perceived false sense of protection against CC has also not been supported by the current literature.^134^ Instead, positive protective health behaviour (e.g. vaccination) could promote participation in future health screenings^135,136^ or alleviate anxieties around screening outcomes, another common barrier to screening.

#### Strength and limitations

This is the first systematic review of barriers, facilitators and factors associated with CC screening for women under 30. Although a previous review has examined reported barriers and facilitators,^17^ this is the first review to also examine factors associated with screening. Moreover, the current review extended the inclusiveness of the original review by including 106 studies across low-, middle- and high-income countries. Title and abstract screening were specifically more inclusive and did not exclude potential studies at this stage, for example due to the lack of mention of the age profile of the sample. A more conservative stance was taken at full-text screening where studies were only included if they explicitly reported personal barriers and facilitators to CC screening in young women aged 25–30. As a result, the current review included 92 studies not included previously and only 14 out of 36 studies from a previous review^17^ were included. Alongside these factors, the current review also implemented a stricter age limit of under 30 compared to under 35.^17^ This allowed for more focused findings for young women and first-time attendees.

The present review also has some limitations. Most included studies were closed-ended cross-sectional surveys. Therefore, the factors highlighted may simply reflect the researcher’s preference and choice of inclusion when designing the surveys. Furthermore, reporting was not always consistent. Barriers and facilitators were often reported without statistical data or weighting, therefore making it difficult to determine the importance or relevance of a factor to the study population. As a result, the current review highlighted the number of times a barrier or facilitator was reported across studies but was unable to determine importance beyond this. Grey literature and unpublished studies were not included in this review and was limited to searching publicly accessible databases only. However, given the size of the review this is unlikely to have changed the main findings.

#### Implications for research and practice

Further research could utilise the key factors associated with screening in young women for targeted interventions to increase and maintain screening uptake. Evidence of an association with vaccination status and screening is highly relevant to the current population. The current review identified that vaccinated women were more likely to attend screening than those who were not vaccinated; however, as the numbers of those vaccinated continue to become more widely available, the impact of vaccination status on CC screening will become more apparent. On an international level, this would be the case for all 27 countries that have introduced HPV vaccination programmes in the last 15 years.^137^ Given that unvaccinated women are less likely to attend screening, vaccination could be further utilised as a facilitator. Therefore, policies and interventions could benefit from promoting HPV vaccination as well as CC screening.

Future research would benefit from implementing a strong theoretical framework, such as the COM-B model of behavioural change,^138^ to categorise and provide further clarity on contributing factors to screening. The COM-B model of behavioural change is designed to provide an overarching framework that captures all factors that influence behaviour change.^138^ The COM-B states that for the behaviour to take place, an individual must (1) have the physical and psychological capability to perform the behaviour; (2) have the physical and social opportunity to do so and (3) have reflective (conscious thought and decision-making) and automatic (habits and subconscious processes) motivation.^138^ Some previous work has utilised the COM-B model when analysing screening behaviours and barriers amongst different age groups.^14,139^ In the current review, reported barriers aligned predominantly with psychological capabilities (e.g. knowledge) and physical opportunity components (e.g. accessibility). The most frequently reported facilitators related to social opportunity (e.g. open communication) and psychological capabilities (e.g. knowledge). Interestingly, factors reflecting psychological capabilities were not prominent in studies that analysed factors associated with screening. Instead, reflective motivational components such as perceived benefits and perceived susceptibility were investigated in some studies and found to be associated with screening attendance, even though they were not frequently reported barriers and facilitators in the included studies.

The lack of attention on psychological factors in studies testing factors associated with screening in young women is surprising given the frequency of these factors as reported barriers. Emotional factors of embarrassment and fear of pain are often reported when discussing screening. When considering external influences that could further contribute to these feelings, healthcare providers should be trained and knowledgeable in in-patient communication to help ease these concerns before, during and after the screening process. Moreover, as negative experiences with CC screening would be limited at this age, compared to older individuals,^140^ the importance of a positive first experience is crucial to ensure that this acts as a facilitator for future attendance. Recommendations from healthcare providers were also one of the most reported facilitators of screening. Healthcare providers can play an active role in the decision to screen but also act as facilitators during screening.

Only one study looked at screening in the LGBTQ+ community and the unique barriers that may impact their screening attendance.^108^ In alignment with an earlier review,^17^ perceptions and screening behaviours of this group are underrepresented in research. Similarly, only two studies from the United States focused on non-native women of this age group. Given the growing number of multicultural populations, particularly in the West, cultural factors must be understood and acknowledged when promoting screening in this age group. Given the importance of communication amongst friends and family as a facilitator to screening, it is important to be aware of differences in taboos and understandings around CC and CC screening.

## Conclusion

The current systematic review highlights several potential factors impacting screening uptake in young women including common barriers of embarrassment, low accessibility and financial constraints, as well as common facilitators such as knowledge, communication and health provider recommendations. In addition, age, marital status, sexual activity and HPV vaccination were shown to be significantly associated with screening uptake. Future research could benefit from adopting stronger theoretical frameworks to categorise and provide further insight into contributing factors affecting screening attendance.

## Supplemental Material

sj-doc-2-whe-10.1177_17455057251324309 – Supplemental material for Identifying the key barriers, facilitators and factors associated with cervical cancer screening attendance in young women: A systematic review Supplemental material, sj-doc-2-whe-10.1177_17455057251324309 for Identifying the key barriers, facilitators and factors associated with cervical cancer screening attendance in young women: A systematic review by Sonia Shpendi, Paul Norman, Jilly Gibson-Miller and Rebecca Webster in Women’s Health

sj-doc-3-whe-10.1177_17455057251324309 – Supplemental material for Identifying the key barriers, facilitators and factors associated with cervical cancer screening attendance in young women: A systematic review Supplemental material, sj-doc-3-whe-10.1177_17455057251324309 for Identifying the key barriers, facilitators and factors associated with cervical cancer screening attendance in young women: A systematic review by Sonia Shpendi, Paul Norman, Jilly Gibson-Miller and Rebecca Webster in Women’s Health

sj-docx-1-whe-10.1177_17455057251324309 – Supplemental material for Identifying the key barriers, facilitators and factors associated with cervical cancer screening attendance in young women: A systematic review Supplemental material, sj-docx-1-whe-10.1177_17455057251324309 for Identifying the key barriers, facilitators and factors associated with cervical cancer screening attendance in young women: A systematic review by Sonia Shpendi, Paul Norman, Jilly Gibson-Miller and Rebecca Webster in Women’s Health

## Declarations

**Ethics approval and consent to participate:** Not applicable as the review is based exclusively on published literature.

**Consent for publication:** Not applicable as the review is based exclusively on published literature.

**Availability of data and materials:** All data and materials included in the submission.
