# Supplementary material for: Identifying the key barriers, facilitators and factors associated with cervical cancer screening attendance in young women: A systematic review
Source: Womens Health (Lond). 2025 Mar 13;21:17455057251324309. doi: 10.1177/17455057251324309 (PMC11907612; doi:10.1177/17455057251324309)
Supplement: sj-doc-2-whe-10.1177_17455057251324309 – Supplemental material for Identifying the key barriers, facilitators and factors associated with cervical cancer screening attendance in young women: A systematic review [file sj-doc-2-whe-10.1177_17455057251324309.doc]

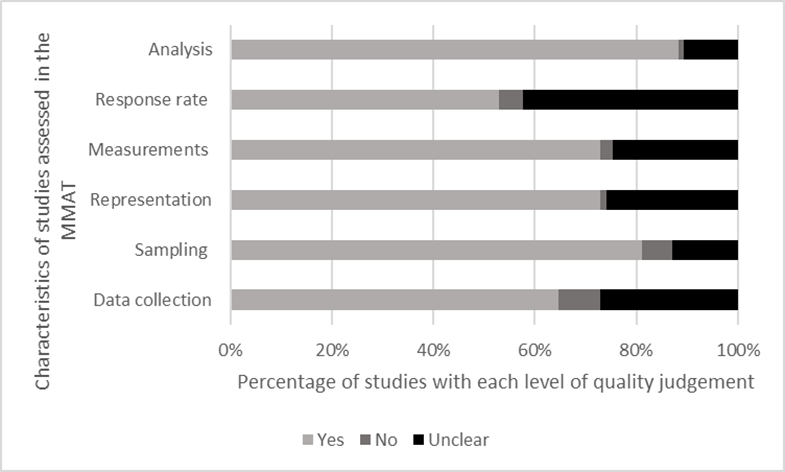


Quality of descriptive quantitative studies (n = 85) *MMAT = Mixed methods appraisal tool


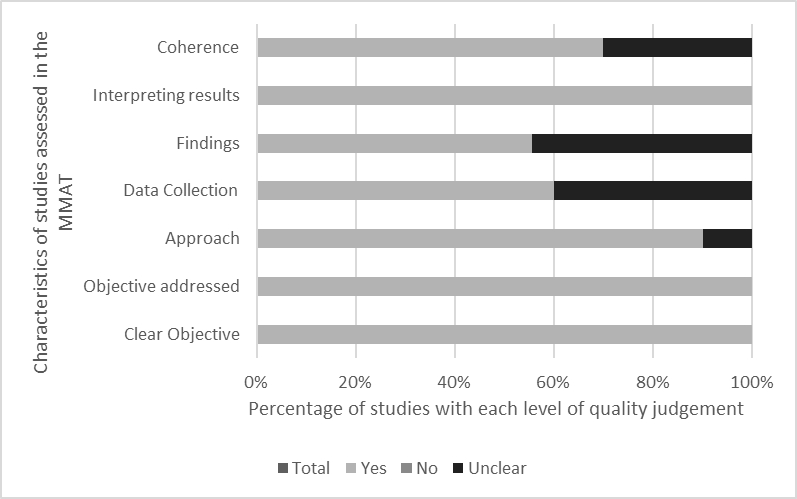


Quality of qualitative studies (*n* = 10) *MMAT = Mixed methods appraisal tool
