# Supplementary material for: Identifying the key barriers, facilitators and factors associated with cervical cancer screening attendance in young women: A systematic review
Source: Womens Health (Lond). 2025 Mar 13;21:17455057251324309. doi: 10.1177/17455057251324309 (PMC11907612; doi:10.1177/17455057251324309)
Supplement: sj-doc-3-whe-10.1177_17455057251324309 – Supplemental material for Identifying the key barriers, facilitators and factors associated with cervical cancer screening attendance in young women: A systematic review [file sj-doc-3-whe-10.1177_17455057251324309.doc]

| **Table 3. Number of participants screened and reported barriers to cervical cancer screening per included study** | | |
| --- | --- | --- |
| **Reference** | **Participants screened for CC (n, %)** | **Barriers (n, %)** |
| Abiodun et al 2014 | EXP 15, 4.3%, CON 12, 3.4% | Lack of awareness, Accessibility constraint, Poor quality of health services, Cost, Lack of interest |
| Abotchie et al 2009 | 13, 12% | Getting cervical test would only make me worry, If I am destined to get cancer, The purpose of screening is to diagnose if I have cancer or not, Screening is not necessary since there is no cure for cancer, The pap test is painful, It is too expensive to have a pap test, It is embarrassing to have a pap test, If a woman is a virgin, pap test will take away her virginity (16, 11.5%), I don't know where I could go if I wanted a pap test, My partner would not want me to have a pap test, If a young woman goes for pap test everyone will think she is having sex |
| Ackerson et al 2008 | 6, NR | Lack of insurance coverage, "Feeling fine" |
| Ackerson et al 2014 | 56, 84% | Lack of knowledge, don’t know where to get appointment, Time-consuming, forget to schedule, Other more important problems, |
| Akpo et al 2016 | 10, 11.24% | Cost, Embarrassing, Feeling "healthy" |
| Akujobi et al 2008 | 0, 0% | Concern of embarrassment of cancer being discovered, Have not heard of it, Do not know where to go for the test, Think it is not necessary, Fear pain and discomfort, Have no time, No reason |
| Al-Naggar et al 2010 | NR, 6% | Pap smear too expensive, Painful, Pap test make me worry, Pap test is embarrassing, Virginity will be taken away if pap test is done to the virgin woman, Do not know where to go if want to have pap smear test, Partner does not allow you to do Pap smear test, Any Health care workers advice you to do the test |
| Alwahaibi et al 2017 | Outpatients NR, 36.8%; Medical Staff NR, 23.3% | Not familiar with/never heard of pap smear, Fearful of pap smear procedures or you had bad experiences when seeing an obstetrician or gynaecologist, Lack privacy, Location for screening is too far, Fearful of dealing with the results, Had no time, Too costly, Not been sexually active for a long period of time, Not married or still young, Ineffective in detecting cervical cancer, Fearful of contracting other diseases, Healthy lifestyle, Religious beliefs / family restrictions, Difficulties to communicate with your gynaecologist,  Others |
| Aniebue et al 2010 | 10, 5.2% | Absence of symptoms, Did not know where to obtain pap smear |
| Anikwe et al 2021 | NR, NR | No time, Fear of result, Don't know, Procedure is cumbersome, Cost consideration, Not sexually active, Not thought of it, Not aware of any screening centre |
| Argaw et al 2022 | 78, 20.3 | No time, "I am not sick", More serious issues to worry about, have not heard of screening, Fear of result, Other |
| Aweke et al 2017 | 58, 9.9% | Not heard of CC, never had an illness, Disease not serious, not aware of CCS, not available nearby, Other |
| Ayeni et al 2023 | 8, 6.9% | Embarrassment, Fear of result, Lack of knowledge, Absence of symptoms, Too expensive, Spouse doesn’t allow it, |
| Ayinde et al 2004 | 35, 8.3% | Lack of awareness about the test, Did not know where to have the test, Cost, Just reluctant to have the test, Belief in not being at risk of cancer of the cervix, Belief that the test is not useful, Feeling that the test is cumbersome, among others |
| Bakogianni et al 2012 | NR, 44.82% | Belief no need for the test, Afraid of visiting a gynaecologist, Quite expensive, If have vaccine do not need pap test |
| Bayu et al 2016 | 235, 19.8% | Feeling "healthy", Cost, Fear of pain, Time consuming, Embarrassment, Time inconvenient |
| Bammeke et al 2014 | NR, NR | Fear of pain, Lack of knowledge/awareness, Not at risk, Accessibility constraints, Don’t know where to get screening, Difficulty getting an appointment, Cost, Haven’t been advised by healthcare worker, Nurses haven’t mentioned screening, Didn’t feel encouraged |
| Black et al 2011 | NR, NR | Moving away from home and not establishing care with a provider or clinic. Difficulty finding a health-care provider, especially female. Fear and discomfort. Lack of education (why testing is important and what it entails). Fear of finding out something is wrong. Test too invasive. Intimidation and embarrassment. Time required. Not owning a car and living in a rural area. Childcare. |
| Burak et al 1998 | 290, 72% | Gynaecological exams were painful or very painful, Embarrassing or very embarrassing, Expensive or very expensive |
| Cooper et al 2018 | NR, NR | Psychological: Self-limiting beliefs, feelings of vulnerability. Physical: "very painful". Time: lack of due to things such as studies, family commitments and wanting to spend time with friends. Location: those who had not didn’t know where and Economic barriers: potential costs. Lack of maternal involvement. |
| Deresse et al 2018 | 124, 15.1% | No screening site, Limited information, too far, no health education programmes to promote, Lack of knowledge of the test, Not engaged in sexually risky behaviour, Cultural beliefs, Spouse doesn't allow, Fear of pain, Shyness, Fear of result, don’t know where to get screening, Other |
| Dhendup et al 2014 | 34, 6% | Never thought that I needed one, embarrassed to get examined by male health professional, Afraid to find out cancer, Wasn’t aware such services are available in our health facilities, Because of past unpleasant experience with health professionals, Others |
| Dozie et al 2021 | 27, 7.2% | Fear of result, Lack of knowledge, Absence of symptoms, Don’t know where to get screening, Cost |
| Easwaran et al 2023 | 8, 5.7% | Embarrassment, Fear of pain/discomfort, Lack of knowledge, Absence of symptoms, |
| Eiser et al 2002 | NR, NR | Worry that the test would be painful, Inconvenience of location |
| Gebisa et al 2022 | NR, 6.3% | Embarrassment, Pain, Feeling "healthy", Spousal constraints, Fear of result, Cost, Lack of knowledge, No reason |
| Gebreegziabher et al 2016 | 24, 10.4% | Carelessness, Fear of pain, Fear of positive result, Embarrassment, not at risk, Lack of time, Expensive cost, Partner influence, don’t where to get screened, don’t know availability, Long distance, Lack of trained nurses, Service inaccessible |
| Gebregziabher et al 2019 | NR, 17.2% | Lack of decision to be screened, Painful, Feel shy |
| Gelassa et al 2023 | 77, 36% | Embarrassment, Pain, Feeling "healthy", Spousal constraints, Afraid of screening, Lack of knowledge, Cost, No reason |
| Getaneh et al 2021 | 2, 0.05% | I am health, It may be painful, I feel shy, not informed about screening place, No interest, partners will not allow, no time, never heard of it |
| Head et al 2012 | 17, 89.5% | Cost, Fear of others finding out, Negative perceptions of screening, Lack of privacy |
| Ibekwe 2015 | DELSUTH 9, 9%; UBTH 7, 7% | Fear of having positive results, Cost of service/lack of money, Because of Shyness, not aware of the Screening centre, No availability of service |
| Ilika et al 2016 | NR, NR | Lack of awareness, Fear of positive result, did not know where to do the screening, Fear of vaginal examination, Fear of false positive screening results, Assumed Pap smear to be harmful, Lack of finances, No reason |
| Isara et al 2013 | 21, 9.1% | No reason was given, Felt it was not necessary to do a Pap smear, Did not know where to go for a Pap smear, Painful, Financial constraints |
| Jemal et al 2023 | NR, NR | “Healthy”, Don’t know where to get screening, Carelessness, Fear of pain, Fear of positive result, Lack of time, Embarrassment |
| Jubelirer et al 1996 | 16, 5.7% | Embarrassment, Pain and discomfort, Fear of finding cancer, Fear of parents discovering sexual activity, Cost, Lack of access to the health care system |
| Kabirir et al 2021 | NR, 21% | Embarrassment of male healthcare worker, Fear of pain, Feeling of shame, Fear of result, Cause of worry, If destined to get cancer then will, Not at risk, Not a priority, Can’t remember to schedule, Busy, Accessibility constraints, Difficulty finding location, Difficulty getting an appointment, Don’t know how to make an appointment, Long waiting hours, Lack of resources/stock, Lack of trained nurses, Cost, Not engaged in sexually risky behaviours, Spouse doesn’t allow it, Too young, For married people, Perception of screening as a negative experience, Fearful of contracting other diseases |
| Kahn et al 1999 | NR, NR | Embarrassment, Pain, Cost, Impulsiveness of plans, Busy, Childcare, Accessibility, Negative perception of screening, Vulnerability, Fear of results, Cancer doesn't impact them, Embarrassment to have a exam by male, "don't want to look for trouble", Lack of knowledge, Fear of parents finding out, Difficulty getting an appointment, Denial, Fear of being diagnosed with pregnancy, Having a medical student present, Peers advised against |
| Kakubari et al 2020 | 141, 22.8% | I do not know what cervical cancer is, I don’t think I am affected with cervical cancer, Okay to get screening years later, I am scared to be diagnosed with CC, For now, I have no symptoms, I am afraid what the cancer screening examination is like, I do not want to go hospital or clinic, I am hesitant to go hospital or clinic, I have not experienced sexual intercourse, I don't want to spend my time for a medical check-up, It bothers me to go to the hospital or clinic, I do not know where to go for cancer screening |
| Karena et al 2024 | 5, 5.2% | Feeling vulnerable, Fear of result, Absence of symptoms, Not at risk, Haven’t decided, Other |
| Lee et al 2017 | NR, NR | Limited knowledge about CC, screening guidelines: attributed to lack of opportunity and/or embarrassment to discuss sex-related matters, and participants belief that they were at low risk of getting CC. more familiar with vaccine than pap smear. Culture-specific barriers: cultural biases towards pap tests and vaccine and prejudice towards women's clinics, specifically gynaecology specialized clinics. Influence of those around them and negative attitudes discouraged participation. Uncomfortable visiting women's clinics. Perception that women’s clinic is only for married women. Seeking gynaecological examinations as a single woman is associated with unacceptable premarital sexual activity or promiscuity. Low accessibility to health care services: Lack of fluency in English. Unfamiliarity with the U.S. health care system, and limited knowledge about health care services for the Pap test and HPV vaccine. |
| Moreira et al 2006 | 166, 81.4% | Embarrassment, Fear of pain, Cannot get an appointment, Do not bother, Other, Embarrassment when undergoing pap smear |
| Moudatsou et al 2022 | 70, 70% | Fear of shame, not sexually active, not necessary, No reason, Other |
| Najem et al 1996 | NR, 55% | Physician did not recommend, lack of awareness of pap test, cost, did not know where to get it, do not think pap test is necessary for them, did not know how to make an appointment, believed they were not at risk for cancer, believed pap test is not accurate, were embarrassed to have the test, did not have time for pap test |
| Natae et al 2021 | 34, 8.7% | Embarrassment, Fear of procedure, Fear of result, Lack of knowledge, Absence of symptoms, Spouse doesn’t allow |
| Ndikom et al 2012 | NR, NR | Cervical cancer screening was never a topic for discussion in the clinic, women are not utilizing it, only the elites utilize it, common belief is that what you don’t know can’t kill you, lack of awareness of cervical cancer and facilities for screening, it is not important to them, nonchalant attitude to one’s health, financial constraints may be the cause sometimes, illiteracy, some people think that such services are for educated people, belief that positive result is death warrant if they are tested positive so it is better not to go for screening, screening services are not easily accessible, poor information dissemination by health workers. |
| Ngari et al 2021 | NR, 18.7% | Lack of knowledge, No interest, Accessibility constraints, Cost, Spouse doesn’t allow it, Religious beliefs, Lack of family support, Cultural biases |
| Ogbonna 2017 | 50, 26.9% | I don’t have time for it, The reception/GP hours are not suitable for me, I am afraid the test will come out positive, I don’t think it is necessary, Not applicable |
| Osei et al 2021 | NR, NR | No sexual activity therefore not eligible, don’t think it's necessary to screen, "I don't have the disease and am not sick", Lack of knowledge about screening type, over 50 years or "older" so will not be affected or motivated to screen, too busy with work to take time off |
| Oshima et al 2013 | 0, 0% | Did not feel “pushed” or encouraged to do so. busy lifestyle. Reluctance to visit the gynaecologist. Unable to speak about such an embarrassing subject with anyone else. Afraid to be seen visiting a gynaecological clinic. Worrying about what others might say about them. Fear or embarrassment (10, NR), the test entailed spreading the legs and exposing oneself. |
| Owoeye 2013 | 22, 12.1% | It is Painful, It is Expensive, It is Embarrassing, I am Healthy, Others, No Response |
| Pegu et al 2017 | NR, NR | Felt that it was not needed, embarrassing, thought it might be costly and painful |
| Reiter et al 2014 | 291, 70% | Cost, embarrassment, lack of a health care provider recommendation, belief pap test is not necessary because they did not have any health problems, All other reasons reported >10%. |
| Rosita et al 2023 | NR, NR | Lack of privacy, Embarrassment of male staff, Time consuming, Lack of interest, Difficulty getting an appointment, Cost, Fear of results, don’t know where to get screening, Lack of knowledge, Spousal constraints |
| Sadler et al 2013 | NR, NR | Lack of knowledge, Fear, Embarrassment, being busy, Difficulty with making appointments, Reduced trust in health recommendations and disgruntlement with previous health care. 'don't feel the need' - especially because young, "related to how at risk you feel". |
| Shin et al 2021 | 0, 0% | Fear of procedure, Embarrassment of male healthcare worker, Lack of awareness, Busy, Worry of what others might say, Fear of parents finding out |
| Singh et al 2022 | NR, 30% | Embarrassment, Fear of procedure, Absence of symptoms, Not at risk, Haven’t been advised by healthcare worker, |
| Singh et al 2012 | 15, NR | No reason, Not feeling at risk, Lack of symptoms, If a woman is a virgin, Pap test will take away her virginity, It is embarrassing to have a Pap test, Afraid of possible outcome, If I am destined to get cancer, Not applicable |
| Tadesse et al 2022 | NR, 2.2% | Lack of knowledge. "healthy", Embarrassment, Cost, No reason |
| Tang et al 1999 | NR, NR | Communication with mother, Openness around sexuality, Prevention orientation, Utilization of Western medicine |
| Tay et al 2015 | 815, NR | People I know do not do it, Embarrassing, Painful, no time, Not having sex. Not necessary. |
| Tesfaye et al 2022 | 120, 30.5% | Fear of result, No reason |
| Thapa et al 2018 | 49, 13.6% | No symptoms, Lack of awareness, Embarrassment, Carelessness, Fear of procedure, financial constraint, Lack of family support, Difficult accessibility, Lack of encouragement from health professional, Uncooperative health professionals |
| Ugonwanyi et al 2014 | 21, 12.2% | Fear of pain, Shyness, Lack of knowledge, Absence of symptoms, Haven't decided, Cost, Other |
| Wellensiek et al 2002 | 148, NR | Did not consider it necessary to have a Pap smear as yet, Had not had a Pap smear because they did not know about it, Afraid or embarrassed, No reason |
| Zaidi et al 2021 | 5, 3.4% | Lack of knowledge, Cost, Loss of Virginity due to test, Fear of genital warts |
| **NR = Not reported EXP = Experimental group CON = Control group DELSUTH = UBTH = DELSUTH = Delta State University Teaching Hospital UBTH = University Benin Teaching Hospital CC = Cervical cancer CCS = Cervical cancer screening** | | |

| **Table 4. Summary of main barriers cited in studies** | |  |
| --- | --- | --- |
| **Barrier theme** | **Specific barriers** | **N** |
| Practical barriers | | |
|  | Financial constraints | 36 |
|  | Accessibility constraints | 34 |
|  | Time constraints | 24 |
| Perceptions of CCS | | |
|  | Embarrassment of procedure | 35 |
|  | Fear of pain/discomfort | 28 |
|  | Embarrassment/fear of result | 24 |
|  | Lack of encouragement to attend | 10 |
|  | Lack of trust in screening | 9 |
|  | Afraid to visit a gynaecologist/doctor | 5 |
| Knowledge misinformation | | |
|  | Attitudes of fatalism | 36 |
|  | Lack of knowledge | 35 |
|  | Lack of interest | 6 |
|  | Not sexually active | 5 |
|  | Loss of virginity due to test | 4 |
| Cultural perceptions | | |
|  | Spouse/Familial constraints | 12 |
|  | Fear of being seen or spoken about | 6 |

| **Table 5.** Number of participants screened and reported facilitators to cervical cancer screening per included study | | |
| --- | --- | --- |
| **Reference** | **Participants screened CC (n, %)** | **Facilitators (n, %)** |
| Ackerson et al 2008 | 6, NR | Influencing individuals were mother, grandparents, friends, and a physician - influenced by her grandmothers’ experiences and wanted to avoid the same thing happening to her. Being vulnerable to cervical cancer was also associated with taking good care of one’s body by getting Pap smears that test for STDs and not having a family history specifically for cervical cancer |
| Alwahaibi et al 2017 | Outpatients NR, 36.8%; Medical Staff NR, 23.3% | Reminded by health care professionals, Understood the importance of screening even when symptoms were absent, Reminded by family or/and friends. Reached the age when performing pap smear is necessary. Funded by the government. Ample time. Family/friends history. Health problems. Others |
| Anaman et al 2017 | 170, 66.9% | Doctors’ recommendation’s, Regular at the doctors, Personally asked for screening, Maternal involvement, Encouragement from family/friends, Community based programme |
| Aniebue et al 2010 | 10, 5.2% | Doctors’ recommendation |
| Bekele et al 2022 | 17, 2.5% | Health worker recommendation, Conversations with friends/relations, Self-conviction |
| Black et al 2011 | NR, NR | Reminded by relative or a health-care provider. Pregnancy, in prison and prompted by health professional, Renewal of oral contraceptives was linked to annual CCS. Assistance with finding physician/health-care provider. Established relationship with a health-care provider or clinic. Education about pap tests (importance and what it entails). Availability of testing by a female provider. Preference for male physician, Event such as "Pap Day" or "Pap Week". Convenience. Friends reminding each other. Reminder and communication methods via email preferred. |
| Cooper et al 2018 | NR, NR | Long term benefit focus: when comparing to negative outcomes of neglecting screening. Maternal involvement: mothers promoting pap smear, as a source of information regarding process and place Friends: friends urging, their previous experiences, discussions less common in those who hadn't and were overdue. Doctor: regular doctor made more comfortable in asking for screening. Previous pap smear facilitator for future pap smears. |
| Easwaran et al 2023 | 8, 5.7% | Previous health problems, Experience of symptoms, Routine |
| Enyan et al 2022 | 21, 4.9% | Routine, Referral, asked for test, Husband's encouragement, Family history, Fear of cancer, heard on the radio, Experience of symptoms, Education from the mosque, Education at the hospital |
| Head et al 2012 | 17, 89.5% | Maternal involvement |
| Kabirir et al 2021 | NR, 21% | Communication with friends, Free, Can afford screening, Awareness, Long-term benefits focus, Health status, Belief reduces risk, Importance of health, Understand the importance of screening, Routine, Youth friendly services |
| Karena et al 2024 | 5, 5.2% | Experience of symptoms, Awareness, Part of general screening programme |
| Osei et al 2021 | NR, NR | Free at the workplace. Free during cervical cancer screening month. Awareness of screening |
| Owoeye et al 2013 | 22, 12.1% | Doctor’s request, Free / Subsidized, Self-conviction, Part of a general screening Program, No response |
| Rosita et al 2023 | NR, NR | Free, Easy access, Encouragement from husband, Encouragement from family, Convenient appointment, Lack of importance |
| Sadler et al 2013 | NR, NR | "Less medical writing" on leaflet. Being told by nurse - ‘actually helped link it  together and stuff, cos otherwise you’d think, I actually thought, it was kind of two different things.’ |
| Tadesse et al 2022 | NR, 2.2% | Health worker recommendation, heard on the news, Communication with friends/family, Information from teacher, Education from religious service, Heard from broacher or other |
| Tay et al 2015 | 815, NR | Belief that screening reduced cervical cancer risk, Screening was recommended by a doctor, Spouse recommended, Familiarity with the test through personal contacts. Someone close to me has done it, Husband said I should do it, People talk about it, Doctor said I should do it, Reduces cervical cancer risk. |
| **NR = not reported STDs = sexual transmitted diseases CCS = cervical cancer screening** | | |

| **Table 6. Summary of main facilitators cited in studies** | |
| --- | --- |
| **Facilitator** | **N** |
| Knowledge and beliefs | 12 |
| Healthcare recommendations and/or reminders | 11 |
| Communication with friends and family | 11 |
| Opportunistic | 8 |

| **Table 7. Number of participants screened and reported factors associated with cervical cancer screening per included study** | | | | |  |  |  |  |
| --- | --- | --- | --- | --- | --- | --- | --- | --- |
| **Reference** | **Participants screened CC (n, %)** | **Factors tested for their association with cervical cancer screening attendance** | **Statistical methods** | **Adjusted variables** |  |  |  |  |
| Ackerson et al 2014 | 56, 84% | **Marital status**, **Smoking status**, **Age at first sex**, Lifetime number of sexual partners, **Perceived benefits**, **Perceived barriers**, Perceived vulnerability | Chi-square and Independent t-tests |  |  |  |  |  |
| Alwahaibi et al 2017 | Outpatients NR, 36.8%; Medical Staff NR, 23.3% | **Marital status**, **Education level** | Chi-square |  |  |  |  |  |
| Alwahaibi et al 2018 | Outpatients NR, 36.8%; Students NR, 23.3% | **Age**, **Marital status**, Education, **Husband's education**, **Family income**, **Family history of cancer**, **Parity**, **Knowledge**, **History of abortion** | Chi-square |  |  |  |  |  |
| Anaman et al 2017 | 170, 66.9% | Age, **Marital status**, Education, **Parity**, **Knowledge**, Religion, **Employment status**, Immigration status, **Perceived susceptibility**, Perceived severity, Health need status, Usual Source of Care, **Consultation with general practitioner** | Bivariate and Multiple logistic regression | All socio-demographic and healthcare characteristics and health-related variables |  |  |  |  |
| Aniebue et al 2010 | 10, 5.2% | **Marital status**, Family income, Smoking history, Sexual activity | Chi-square |  |  |  |  |  |
| Anikwe et al 2021 | NR, NR | Age, **Marital status**, Parity, Religion, Social class, Ethnicity | Chi-square |  |  |  |  |  |
| Annan et al 2019 | NR, NR | **Screening/CC knowledge**, Perceived susceptibility, **Perceived severity**, perceived logistical barriers, Benefits-minus-barriers | Pearson's correlation and Mediation analysis | CC knowledge, perceived susceptibility, seriousness, benefits, barriers |  |  |  |  |
| Argaw et al 2022 | 78, 20.3 | **Age**, Marital status, **Knowledge,** Smoking history, Lifetime number of sexual partners, STI | Multivariate binary logistic regressions | NR |  |  |  |  |
| Aweke et al 2017 | 58, 9.9% | Age, Education, Income, Knowledge, **ever received information**, Religion, Husband's education, **Health seeking behaviour** | Multivariate logistic regression | Knowledge score, ever received information and active health information seeking |  |  |  |  |
| Ayinde et al 2004 | 35, 8.3% | **Marital status**, **Education**, Religion, Place of residence, **Sexual activity** | Chi-square |  |  |  |  |  |
| Bayu et al 2016 | 235, 19.8% | Age, Marital status, Employment status, Pregnancy, Household income, Age at first sex, **Lifetime number of sexual partners**, **History of STI**, **History of HIV**, Type of birth/delivery, **Knowledge**, **Perceived susceptibility**, Perceived severity **Perceived barriers** | Bivariate and Multiple logistic regression | Age, history of multiple sexual partners, history of sexually transmitted disease, HIV Sero status, overall knowledge of cancer and screening, perceived susceptibility to develop cancer and perceived barriers |  |  |  |  |
| Beer et al 2014 | 14 164, 45.9% | Age, **Deprivation scale**, Maternal age, Childhood vaccination status, **HPV vaccination** | Univariate binary logistic regression and Multivariate binary logistic regression | Quintile of social deprivation, maternal age at birth, gestational age at birth, childhood vaccination |  |  |  |  |
| Bekele et al 2022 | 17, 2.5% | **Education**, Marital status, **Attitude**, **Knowledge** | Bivariate and Multiple logistic regression |  |  |  |  |  |
| Binka et al 2016 | NR, 8% | Age, Martial status, Religion, **Employment status** | Binary logistic regression |  |  |  |  |  |
| Boone et al 2016 | Unvac NR, 51%; Vacc NR, 59% | **Age**, **HPV vaccination**, **Screening initiation**, **Ethnicity** | Conditional cox proportional hazards regression models stratified by matched pairs (Hazard ratios) | Age, initial screening age, and race |  |  |  |  |
| Budd et al 2014 | NR, NR | **Age**, **HPV vaccine** | Chi-square |  |  |  |  |  |
| Burak et al 1998 | 290, 72% | Perceived susceptibility, **Cues to action**, Perceived severity, **Benefits-minus-barriers** | Multiple regression analysis and Independent t-tests |  |  |  |  |  |
| Byrd et al 2004 | NR, 69% | **Acculturation**, **Insurance status**, Education, **Perceived Severity**, **Perceived barriers** | Bivariate and Multivariate logistic regression | Acculturation level, belief that the Pap test would be painful, not knowing where to go for a Pap test, and believing that most unmarried young women go for Pap tests |  |  |  |  |
| Changkun et al 2022 | NR, 21.6% | **Age**, Education, **Employment status**, **Insurance status**, **Media use/exposure**, **Religion**, **Wealth index**, **Head of household sex**, **Husband education**, **Place of residence**, **Husband/Partner role** | bivariable and Multivariable regression models | NR |  |  |  |  |
| Chao et al 2017 | NR, NR | Education, **Income**, **Pregnancy**, **HPV vaccination**, **Ethnicity**, **Routine hospital visit**, **STI**, Insurance status, **Abnormal pap result** | Bivariate and Multivariable Cox models | Race/ethnicity, census block income and education level, percent of adults in the census block with high school degree or higher, Medicaid enrolment, mean length of membership, primary care provider characteristics, healthcare use within 12 months before baseline, flu vaccination within 12 months before baseline, Gynecologic history before baseline, Human papillomavirus vaccination history before baseline |  |  |  |  |
| Dhendup et al 2014 | 34, 6% | **Age**, **Martial status**, Country of graduation, **Screening/CC knowledge**, **Recommended during last visit** | Cross-tabulation and Fischer Exact test |  |  |  |  |  |
| Dozie et al 2021 | 27, 7.2% | **Age**, **Marital Status**, **Education**, **Income** | Chi-square |  |  |  |  |  |
| Easwaran et al 2023 | 8, 5.7% | Education, Family history of cancer | Chi-square |  |  |  |  |  |
| Enyan et al 2022 | 21, 4.9% | Marital status, Insurance status, Employment status, **Income**, **Education**, Modesty, Religion, Self-sampling, **Knowledge**, Attitude, Cultural barriers, Perceived behavioural control | Chi-square test, Point biserial correlation and Binary logistic regression analysis |  |  |  |  |  |
| Gebisa et al 2022 | NR, 6.3% | **Knowledge**, **Attitude** | Binary logistic regression and Multivariate logistic regression analysis | NR |  |  |  |  |
| Gebreegziabher et al 2016 | 24, 10.4% | Age, Marital status, Education, **Workplace**, Service years, Ethnicity, Parity, Knowledge, **Attitude** | Bivariate and Multivariate logistic regression analysis | NR |  |  |  |  |
| Gebregziabher et al 2019 | NR, 17.2% | **Age**, **Marital status**, **Education**, **Birthplace**, **Sexual activity** | Binary logistic regression and Multivariate logistic regression analysis | Age, sexual experience, marital status, place of birth, level of education |  |  |  |  |
| Gebru et al 2016 | NR, NR | **Age**, **Income**, **Parity**, Perceived susceptibility, Perceived severity | Bivariate and Multivariate logistic regression analysis | Parity, marital age, average monthly income, age group, educational status, perceived severity to cervical cancer and perceived benefits of cervical cancer screening |  |  |  |  |
| Gelassa et al 2023 | 77, 36% | **Education**, Use of family planning services, Abortion, **know someone with CC**, **Knowledge**, **Feeling at risk**, **Information on CC** | Bivariable logistic regression and Multivariable logistic regression | Educational status, use of family planning, history of abortion, having knowledge about cervical cancer, knowing someone diagnosed with cervical cancer, the source of information and the number of sexual partners |  |  |  |  |
| Guo et al 2017 | Unvac NR, 75.6%; Vacc NR, 88.4% | **Age**, **Education**, **HPV vaccination**, **Ethnicity**, **Place of residence**, **Immigration status**, **Having a usual source of care**, **Routine check-up by OB/GYN in past year**, **Insurance status** | Multivariate logistic regression | Age, race/ethnicity, region of residence, insurance type, and education level |  |  |  |  |
| Hauwa et al 2021 |  | **Education**, **Income**, Screening & CC Knowledge, **Religion, Attitude**, **Availability** | Chi-square |  |  |  |  |  |
| Hirth et al 2016 | 19 797, 79.3% | **Age**, **HPV vaccination**, **Vaccination provider**, **Time of vaccination doses**, Place of residence, **Usual source of care**, **History of cancer**, **Paediatric provider** | Multivariate binary logistic regression |  |  |  |  |  |
| Hoque et al 2014 | 22. 15% | **Screening and CC knowledge**, HBM constructs, Perceived susceptibility, **Perceived logistical barriers**, **Self-efficacy**, **Benefit-minus-barriers** | Pearson correlation test and T-test |  |  |  |  |  |
| Ilika et al 2016 | NR, NR | Age | Chi-square and Independent t-tests |  |  |  |  |  |
| Isabirye et al 2022 | 1 338, 13.4% | **Age**, **Religion**, **Education**, **Employment**, **Insurance status**, **Place of residence**, **Contraception**, **Gravidity**, **Age at first sex**, **Wealth index**, **Media exposure**, **Marriage type**, **Household size**, **Birthplace**, **Attitudes towards IPV against women**, **Decision making autonomy**, **Household wealth index**, **Accessibility to healthcare** | Chi-square and Cross-tabulation |  |  |  |  |  |
| Isara et al 2013 | 21, 9.1% | Screening & CC knowledge | Cross-tabulation and Fischer Exact test |  |  |  |  |  |
| Jemal et al 2023 | NR, NR | Age, Marital status, **Education**, **Parity, Knowledge,** Employment status, **Number of sexual partners** | Binary logistic regression and Multivariate logistic regression analysis | Age, marital status, educational  status, service area, working experience, history of multiple sexual partners, parity, comprehensive knowledge of cervical cancer and its screening method |  |  |  |  |
| Kabirir et al 2021 | NR, 21% | **Marital status**, **Education**, Religion, **Lifetime number of sexual partners**, Age at first sexual activity, **Sexual activity, Use of family planning services, Willingness to screen** | Chi-square |  |  |  |  |  |
| Kakubari et al 2020 | Unvac 52, 17%; Vacc 89, 29% | **HPV vaccination** | Fisher's exact test |  |  |  |  |  |
| Kaneko 2018 | 383, NR | **Age**, Education, **Income**, Screening/CC Knowledge, **Employment status**, **HPV vaccination**, Routine hospital visit, Smoking history, **Lifetime number of sex partners**, **Concerns regarding STIs**, Intervention, **Perceived susceptibility**, **Perceived logistical barriers**, **Confidence of undergoing screening with male physician**, Insurance status, Receipt of coupon for smear from government | Chi-square and Univariate analysis | Age, employment status, income |  |  |  |  |
| Kim et al 2016 | 10 204, 100% | **Age, HPV vaccination** | Chi-square and Logistic regression | Neighbourhood income, urban versus rural residency, the laboratory service |  |  |  |  |
| Kitchener et al 2018 | NR, NR | **HPV vaccination**, **Region** | Logistic generalised estimating equations model | Baseline uptake rate, intervention and baseline practice rate, practice attendance and PCT region |  |  |  |  |
| Kreusch et al 2018 | 183 655, 70.2% | **HPV vaccination**, **Education**, Income, Migration, Screening invitation | Cox regression and explorative model building | Education, income and migration history |  |  |  |  |
| Langille et al 2006 | 246, 45% | **Age**, Education, Parent's education, Parent's employment, **Place of residence**, **Seeing family as advantaged**, **Contraception** | Chi-square and Stepwise logistic regression | Grade and school, and incorporating a grade-by-school interaction term |  |  |  |  |
| Lee et al 2015 | 123, 75% | Age, **Marital status**, Education, Screening/CC knowledge, Employment status, Self-efficacy, Modesty, Fatalism, Health needs status, Insurance status | Chi-square, T-tests and Hierarchical binary logistic regression |  |  |  |  |  |
| Letuka et al 2018 | 50, 15.2% | **Marital status**, **Education**, Household wealth index, **Place of residence**, Age at first sexual activity | Chi-square, Cross-tabulation and Binary logistic regression model | NR |  |  |  |  |
| Mather et al 2012 | 30, 48% | Age, Parents education, Family history of cancer, Religion, Importance of religion, HPV vaccination, Ethnicity, Sexual activity, Screening invitation, | Multiple linear regression and Logistic regression | Age and eligibility for screening |  |  |  |  |
| Miyoshi et al 2021 | NR, 12.6% | **HPV vaccination** | Chi-square and Logistic regression |  |  |  |  |  |
| Moudatsou et al 2022 | 70, 70% | **Education** | Chi-square and Independent t-test |  |  |  |  |  |
| Mpachika-Mfipa et al 2023 | 63, NR | **Knowledge, Source of information, Beliefs of CC,** Screening location, Lifetime sexual partners, **History of HIV, Willingness, Physician recommendation** | Chi-square |  |  |  |  |  |
| Mpachika-Mfipa et al 2022 | 63, NR | **Age,** Marital status, **Religion**, **Ethnicity**, **Place of residence** | Bivariate logistic regressions and Multivariable logistic regression | Age-group, religion, ethnicity, marital status and area of residence |  |  |  |  |
| Najem et al 1996 | NR, 55% | **Age**, **Family income**, **Knowledge**, **Ethnicity**, **Smoking history**, **Sexual activity**, **Physicians recommendations**, **Previous screening by family member** | Logistic regression and Multivariate logistic regression | NR |  |  |  |  |
| Natae et al 2021 | 34, 8.7% | **Age**, **Parity, Knowledge of screening,** Knowledge of CC**, Availability of screening, Routine check-up with OB/GYN in past year, History of cancer** | Binary logistic regression and Multivariate logistic regression | Age, knowing the availability of the screening service in the public hospital, history of early sexual initiation, know the consequence of advanced cervical cancer (metastasis and bleeding), knowledge of CC screening, and discussion on cervical cancer with healthcare providers |  |  |  |  |
| Ogbechie et al 2012 | NR, 93.3% | **STI testing** | Fisher's exact test |  |  |  |  |  |
| Owoeye et al 2013 | 22, 12.1% | **Awareness** | Chi-square and Independent t-test |  |  |  |  |  |
| Park et al 2023 | 8,149, 46% | Age, **Employment status**, **Immigration status**, **Nationality**, **Place of residence**, **Deprivation scale**, **Comorbidity**, Consultation with general practioner | Chi-square and Independent t-test and Multiple logistic regression | Age, duration of stay, nationality, residence, economic status, occupation, and comorbidity |  |  |  |  |
| Paynter et al 2015 | 1 276, 55% | **Age, HPV vaccine**, **Parity**, **Gravidity**, **Ethnicity** | Binary logistic regression | At least one HPV4 dose, number of doses, race, gravidity and parity |  |  |  |  |
| Pengpid et al 2014 | NR, 11.6% | **Marital status**, **Household wealth index**, **Place of residence**, **Attitude**, **Smoking history**, **Lifetime number of sexual partners**, **Sexual activity**, **Sexual experience**, **STI**, **Benefits-minus-barriers** | Logistic regression |  |  |  |  |  |
| Reiter et al 2014 | 291, 70% | **Age**, **Marital status**, Education, Income, **HPV knowledge**, Employment, **HPV vaccination**, **Ethnicity**, Urbanicity, **Lifetime number of sexual partners**, **Age at first sexual activity**, **Sexual identity**, **STI**, Worry of HPV-related disease, Perceived susceptibility, Perceived severity of HPV-related disease, **Perceived lower risk of CC compared to heterosexual women**, **Routine check-up by OB/GYN in past year**, **Insurance status**, **Disclosed sexual orientation to healthcare provider**, Discriminated against by healthcare provider | Univariate and Multivariate logistic regression and Chi-square | NR |  |  |  |  |
| Sauer et al 2015 | 6 023, 82.6% | **Age**, **Education**, Income, **HPV vaccination**, Ethnicity | Adjusted predicted marginal models | Age, race/ethnicity, education, income, immigration status, smoking status, health insurance status, having a medical home, having had a recent healthcare visit, and geographic region |  |  |  |  |
| Sauvageau et al 2021 | 871, 59% | HPV vaccination | Chi-square, Fisher's exact test and Log binomial regression | Age, ethnicity, use of contraception, having a family physician, level of knowledge about STI and number of sexual partners during the life |  |  |  |  |
| Seay et al 2022 | NR, NR | **Age**, Ethnicity, Marital status, Education, Mental health, **Deployment status (Army)**, **Employment status**, Income | Generalized estimating equations | NR |  |  |  |  |
| Shand et al 2010 | NR, 63% | **Marital status**, Family history of cancer, **Sexual activity**, **Abnormal pap result** | Chi-square and t-test statistics |  |  |  |  |  |
| Shin et al 2022 | 1,533, 39.1% | Age, Marital status, Number of births, **Maternal age** | Chi-square, Simple logistic regression and Multiple logistic regression | Age, self-rated health status, supplemental medical insurance for cancer, education level, monthly household income, and family member count |  |  |  |  |
| Singh et al 2012 | 15, NR | **Marital status** | Chi-square |  |  |  |  |  |
| Tang et al 1999 | NR, NR | Age, **Education**, Parents education, Family history of cancer, Health professionals in family, **Ethnicity**, **Cultural barriers**, **Acculturation**, **Sexual experience**, Sexual identity, Communication with mother, **Benefits-minus-barriers**, Usual source of care, **Health history**, **Routine check-up by OB/GYN in past year,** **General demographics** | Logistic regression and T-test | Race |  |  |  |  |
| Tay et al 2015 | 815, NR | **Age**, Screening/CC knowledge, Perceived susceptibility, Usual source of care, **Specialist consultation within past 3 years**, **Routine check-up by OB/GYN in past year** | Chi-square |  |  |  |  |  |
| Tesfaye et al 2022 | 120, 30.5% | **Age**, **Employment status**, **Source of information from health worker**, Source of information from printed materials, **Lifetime number of sexual partners**, **History of an STI**, **Knowledge**, **Attitude** | Multiple logistic regression analysis and Backward stepwise selection | Age of the women, adherence supporter, and source of information from health care professionals, history of multiple sexual partners, sexually transmitted infection, having high knowledge score and high attitude score |  |  |  |  |
| Thapa et al 2018 | 49, 13.6% | Age, **Marital status**, **Education**, **Ethnicity**, **Family history of cancer**, Knowledge. Attitude | Cross-tabulations and Binary logistic regression |  |  |  |  |  |
| Ugonwanyi et al 2014 | 21, 12.2% | **Perception of screening** | Chi-square, T-test and Multivariate analysis | NR |  |  |  |  |
| Wellensiek et al 2002 | 148, NR | **Education**, **Parity**, **Smoking history**, Age at first sexual activity, Sexual experience | Chi-square and Fisher's exact test |  |  |  |  |  |
| Yi 1998 | 74, 36.8% | **Marital status**, Income, **Religion**, Birthplace, **Acculturation**, **Sexual activity**, **Usual source of care**, Insurance status | Chi-square, T-test and Bivariate logistic regression |  |  |  |  |  |
| Yoo et al 2011 | NR, 48% | **Age**, Screening/CC knowledge, HPV knowledge, **Ethnicity**, **Birthplace**, **Comfort with pap test** | Logistic regression |  |  |  |  |  |
| Zaidi et al 2021 | 5, 3.4% | **Knowledge** | Univariate and Multivariate regression | Age, year of study, family income, HPV vaccinated |  |  |  |  |
| Bold = significant association NR = not reported Vacc = HPV vaccinated Unvac = not HPV vaccinated HPV = human papillomavirus CC = cervical cancer STI = sexually transmit infection HIV = human immunodeficiency virus OB/GYN = obstetrics and gynaecology IPV = intimate partner violence PCT = primary care trust | | | | |  |  |  |  |

| **Table 8. Summary of main factors associated with screening cited in studies** | | | |  |
| --- | --- | --- | --- | --- |
| **Factors associated theme** | **Factors associated** | **Total N** | **Significant N** | **Direction** |
| Socio-demographic factors | | | |  |
|  | Age | 31 | 20 | Older |
|  | Marital status | 28 | 18 | Married |
|  | Education | 28 | 16 | Higher level |
|  | Knowledge | 24 | 16 | Higher scores |
|  | Employment status | 14 | 9 | Employed |
|  | Sexual activity | 13 | 8 | Active |
|  | Ethnicity | 10 | 7 | Mixed |
|  | Lifetime sexual partners | 10 | 7 | More partners |
|  | Parity | 9 | 7 | More children |
|  | Income | 9 | 5 | Higher-income |
|  | Religion | 12 | 5 | Mixed |
|  | History of STI | 7 | 4 | Yes |
|  | Place of residence | 9 | 6 | Urban |
|  | Smoking status | 6 | 3 | Smokers |
| Vaccination status | | | |  |
|  | HPV vaccinated | 12 | 11 | Vaccinated |
|  | Age and vaccination status | 7 | 6 | Vaccinated |
|  | Race and vaccination status | 3 | 3 | Vaccinated |
| Psychological factors | | | |  |
|  | Perceived susceptibility | 11 | 5 | Higher scores |
|  | Perceived benefits and/or prevention orientation | 6 | 5 | Higher scores |
|  | Perceived logistical barriers | 6 | 5 | Fewer |
| Previous experience | | | |  |
|  | Insurance status | 9 | 4 | Insured |
|  | Routine check-up/visited gynaecologists | 5 | 5 | Yes |
| Note: Yes = Yes to a history of STI & Yes to routine check-ups | | | | |
